# Early prediction of neurological outcome after barbiturate coma therapy in patients undergoing brain tumor surgery

**DOI:** 10.1371/journal.pone.0215280

**Published:** 2019-04-17

**Authors:** Jeong-Am Ryu, Wonkyung Jung, Yoo Jin Jung, Do Yeon Kwon, Kina Kang, Hyeok Choi, Doo-Sik Kong, Ho Jun Seol, Jung-Il Lee

**Affiliations:** 1 Department of Critical Care Medicine, Samsung Medical Center, Sungkyunkwan University School of Medicine, Seoul, Republic of Korea; 2 Department of Neurosurgery, Samsung Medical Center, Sungkyunkwan University School of Medicine, Seoul, Republic of Korea; 3 Department of Nursing, Neurosurgery Intensive Care Unit, Samsung Medical Center, Seoul, Republic of Korea; George Washington University, UNITED STATES

## Abstract

After a difficult brain tumor surgery, refractory intracranial hypertension (RICH) may occur due to residual tumor or post-operative complications such as hemorrhage, infarction, and aggravated brain edema. We investigated which predictors are associated with prognosis when using barbiturate coma therapy (BCT) as a second-tier therapy to control RICH after brain tumor surgery. The study included adult patients who underwent BCT after brain tumor surgery between January 2010 and December 2016. The primary outcome was neurological status upon hospital discharge, which was assessed using the Glasgow Outcome Scale (GOS). In the study period, 4,296 patients underwent brain tumor surgery in total. Of these patients, BCT was performed in 73 patients (1.7%). Among these 73 patients, 56 (76.7%) survived to discharge and 25 (34.2%) showed favorable neurological outcomes (GOS scores of 4 and 5). Invasive monitoring of intracranial pressure (ICP) was performed in 60 (82.2%) patients, and revealed that the maximal ICP within 6 h after BCT was significantly lower in patients with favorable neurological outcome as well as in survivors (*p* = 0.008 and *p* = 0.028, respectively). Uncontrolled RICH (ICP ≥ 22 mm Hg within 6 h of BCT) was an important predictor of mortality after BCT (adjusted hazard ratio 12.91, 95% confidence interval [CI] 2.788–59.749), and in particular, ICP ≥ 15 mm Hg within 6 h of BCT was associated with poor neurological outcome (adjusted odds ratio 9.36, 95% CI 1.664–52.614). Therefore, early-controlled ICP after BCT was associated with clinical prognosis. There were no significant differences in the complications associated with BCT between the two neurological outcome groups. No BCT-induced death was observed. The active and timely control of RICH may be beneficial for clinical outcomes in patients with RICH after brain tumor surgery.

## Introduction

Increased intracranial pressure (ICP) generally occurs in patients with brain tumor because of tumor-associated brain edema, tumor per se, or tumor bleeding [1]. Peritumoral edema is a leading cause of morbidity and mortality in patients with brain tumors [2]. Uncontrolled cerebral edema may result in refractory intracranial hypertension (RICH), and also leads to severe neurological deficits and potentially fatal herniation [1,3]. Therefore, treatment for RICH entails medical or surgical interventions. Even when brain tumors are surgically resected, patients should be monitored vigilantly in the intensive care unit, because aggravated brain swelling after tumor resection is not uncommon [1]. In addition, RICH may be caused by residual tumor or post-operative complications such as hemorrhage, infarction, and aggravated brain edema. Therefore, the management of RICH and brain edema are crucial issues in patients undergoing brain tumor surgery. Barbiturate coma therapy (BCT) is currently used as a second-tier therapy to control RICH, and it has been shown to be associated with potential benefits in traumatic brain injury (TBI) or malignant infarction [2,4,5]. Therefore, BCT may be helpful for controlling RICH after brain tumor surgery. However, there have been limited reports on BCT after brain tumor surgery. In addition, it is unknown which factors are important for prognosis if BCT is performed after tumor surgery. The objective of this study was to investigate which predictors are associated with clinical outcomes when BCT is used as a second-tier therapy to control RICH after brain tumor surgery.

## Materials and methods

### Study population

**Fig 1:**
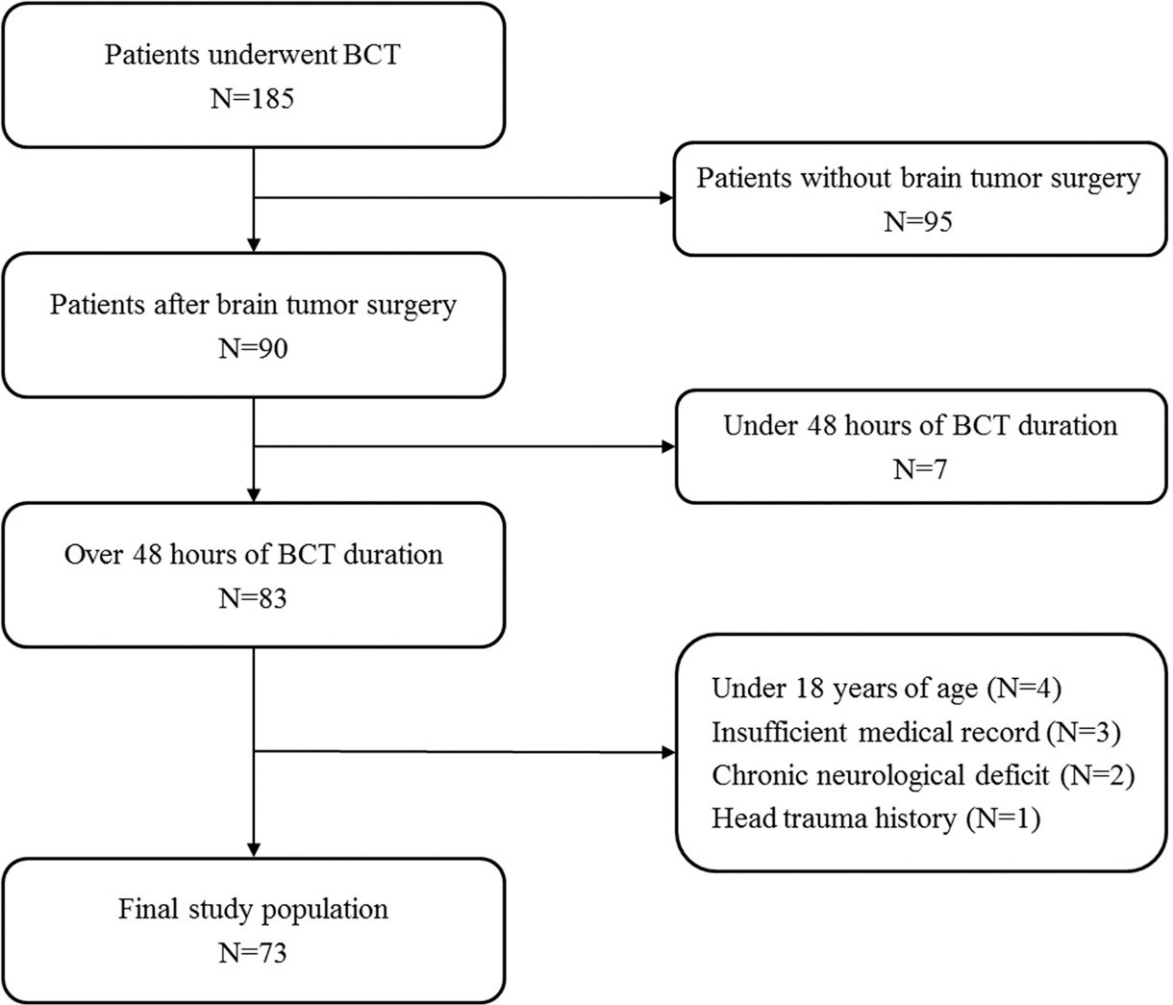
Study flow chart. BCT, barbiturate coma therapy.

This was a retrospective and observational study of adult patients who underwent BCT after brain tumor surgery while being hospitalized at Samsung Medical Center between January 2010 and December 2016. This study was approved by the Institutional Review Board of Samsung Medical Center (SMC 2017-11-141). The requirement for informed consent was waived due to its retrospective nature. Clinical and laboratory data were collected by a trained study coordinator using a standardized case report form. We included patients who underwent BCT after brain tumor surgery during the study period. BCT was determined by a neurosurgeon according to the BCT protocol: BCT was performed in 1) patients with impending or progressive clinical signs of herniation, such as abnormal pupillary reflex, absent doll’s eye reflex, or abnormal posturing; 2) patients with significantly progressive mass effect or suspected herniation radiologically consistent with severe neurological deterioration; and 3) patients who had RICH or were suspected to have RICH, as determined by a neurosurgeon during surgery. We excluded patients who were aged less than 18 years, those who received less than 48 h of BCT, those with insufficient or incomplete medical records, and those who had a history of head trauma or chronic neurological abnormality (Glasgow Outcome Scale [GOS] ≤3) following admission to the intensive care unit (ICU). In total, 73 patients who underwent BCT after brain tumor surgery were analyzed in this study (Fig 1).

### Definitions and outcomes

In this study, RICH was defined as an ICP of 21 to 29 mm Hg for 30 min or more, an ICP of 30 to 39 mm Hg for 15 min or more, or an ICP greater than 40 mm Hg for more than 1 min, in the absence of external interventions [6]. Uncontrolled RICH was defined as a maximal ICP of 22 mm Hg or more occurring within the initial 6 h of barbiturate infusion [5,7]. The ICP was monitored using an external ventricular drain, an intraparenchymal fiber optic sensor (Camino Laboratories, San Diego, California, USA), or an epidural pneumatic sensor (Spiegelberg GmbH & Co. KG, Hamburg, Germany). Primary brain tumors were classified and graded according to the 2016 World Health Organization Classification of Tumors of the Central Nervous System [8]. The computed tomography (CT) scorings at that time of BCT were performed using the Rotterdam CT scan scoring system [9]. The severity of illness was assessed by the Sequential Organ Failure Assessment (SOFA) scores and the Acute Physiology and Chronic Health Evaluation II (APACHE II) scores [10–12]. The primary outcome was neurological status upon hospital discharge, as assessed by GOS (1 to 5) [13]: GOS scores of 4 and 5 were considered to reflect favorable neurological outcomes, while GOS scores of 1, 2, and 3 were considered to reflect poor neurological outcomes. We thoroughly reviewed each patient’s medical records. Patients were independently graded on the GOS by a neurointensivist and a physiatrist. The secondary outcome was in-hospital mortality. All recorded brain CT scans in this study were taken within 6 h prior to BCT. For all CT studies, 64-channel scanners (Light Speed VCT; GE Healthcare, Milwaukee, Wisconsin, USA) with a slice width of 5-mm were used. The clinical information collected daily included the use of vasoactive medications, blood tests, and highest ventilator settings. Acute lung injury was defined as a PaO_2_/FiO_2_ ratio under 300. Severe hypoxia was defined as a PaO_2_/FiO_2_ ratio under 200 [5]. Ventilator-associated pneumonia (VAP) was defined as a new infiltrate on chest radiograph with clinical signs during mechanical ventilation [5]. The nosocomial infections recorded included bacteremia, urinary tract infections, and gastrointestinal infections. Acute kidney injury was defined by creatinine levels over 176.8 μmol/L, or twice the initial baseline level. Liver dysfunction was defined by a total bilirubin level exceeding 34.2 μmol/L in the absence of chronic liver diseases. Body temperature was measured using an ear thermometer. Hypothermia was defined as a body temperature under 35°C [14].

### Barbiturate coma therapy

Pentobarbital was administered in accordance with the protocol established by the critical pathway of BCT of the Samsung medical center [15]. We assessed each patient’s clinical status prior to initiating BCT. Ventilatory status was secured by mechanical ventilation. SaO_2_ exceeded 95%, and CO_2_ was titrated to control ICP using End Tidal CO_2_ as a guide. Euvolemic state was initially targeted; central venous pressure was maintained from 8 to 12 mm Hg titrated with 5% albumin or normal saline. The initial goal of cerebral perfusion pressure was over 60 mm Hg. Vasopressors and/or inotropics were administered in order to achieve adequate cerebral perfusion pressure in spite of initial volume management, as stated above. BCT was initiated by intravenously administering pentobarbital at a loading dose of 10 mg/kg for 60 min (bolus injection of 2.5 mg/kg every 15 min) followed by a continuous infusion of 10 mg/kg per h for 3 h, which was then followed by a maintenance dose of 1.5–3 mg/kg per h. Bispectral Index (BIS) or continuous electroencephalography (cEEG) was monitored in order to ensure the titration of an adequate maintenance dosage. The optimal maintenance dosage was continuously titrated for a burst suppression of 10 to 12 s on cEEG, or the simultaneous achievement of a score below 20 on the BIS and concurrent suppression ratio (SR) over 60%. If the initial targets of cEEG or BIS were attained, neurological examination was repeated so as to evaluate the patient’s neurological state with a focus on possible changes in brainstem reflex, size-varying and nonreactive pupils, and diminished blink and cough reflexes. If the patient was deemed to be neurologically stable, the pentobarbital infusion rate was maintained for a BIS score between 10 to 20 and over 60% of SR. We followed up with brain CT and re-evaluated the need for surgical decompression. If the ICP was stable for more than three to five days, the pentobarbital dosage was gradually reduced at a rate of 0.5 mg/kg per 4 h, then tapered out. No neuromuscular blockers were used.

### Statistical analyses

All data were presented as medians and interquartile ranges (IQRs) for continuous variables and as numbers (percentages) for categorical variables. Data were compared using the Mann-Whitney U test for continuous variables and the Chi-squared test or Fisher’s exact test for categorical variables. The Wilcoxon signed-rank test was used to compare paired maximal/mean ICPs before and after BCT. The Mann-Whitney U test was used to compare the maximal ICPs after BCT between favorable and poor neurological outcomes as well as between survivors and non-survivors. The predictive performance of the maximal ICPs after BCT was assessed using area under the curve (AUC) of the receiver operating characteristic (ROC) curves for sensitivity vs. 1-specificity. The optimal cut-off value of the ICP level for the prediction of poor neurological outcome was obtained by the ROC curve and Youden index [16,17]. The Kaplan-Meier method was used to generate survival curves between survivors and non-survivors, which were compared using the log-rank test. The Cox proportional hazards model was used to analyze the effects of covariates on survival times with estimated hazard ratios (HR) with 95% confidence intervals (CIs). Multivariable logistic regression analysis was used to identify the independent predictors of poor neurological outcome in patients who underwent BCT with estimated odds ratios (OR) and 95% CIs. Variables with *p*-values under 0.2 in univariate analyses and clinically relevant variables were subjected to a stepwise multiple logistic regression model in order to obtain the final parsimonious model. All of the tests were two-sided, and in all tests p values < 0.05 were considered to be statistically significant. All data were analyzed using the Statistical Package for the Social Science software version 20.0 (IBM, Armonk, NY, USA).

## Results

### Baseline characteristics and clinical outcomes

Throughout the study period, 4,296 patients underwent brain tumor surgery. Of these 4,296 patients, 73 underwent BCT (1.7%). The median age of the patients who underwent BCT was 48 years (IQR 38–56 years). Thirty-seven (50.7%) out of these 73 patients were male. Seventy (95.9%) of the patients had primary brain tumors while the remaining 3 (4.1%) had metastatic brain tumors. The most common tumor was meningioma (37.0%), followed by diffuse astrocytic and oligodendroglial tumors (27.4%). However, there was no significant difference in the final histopathological classification of brain tumor between the two groups with different neurological outcomes (*p* = 0.124). Supratentorial origin (69.9%) was the most common origin. Hypertension (17.8%) and diabetes mellitus (8.2%) were the most common comorbidities. BCT was performed immediately after tumor surgery in 47 patients (64.4%). The baseline characteristics of the patients are presented in Table 1. Invasive ICP monitoring was performed during BCT in 60 (82.2%) patients. There were no significant differences in gender, comorbidities, grading of brain tumors, initial methods of ICP management, initial brain CT findings, and GCS, or APACHE II and SOFA scores on BCT between the two groups with different neurological outcomes. Prior to BCT, hyperosmolar therapy was performed in most patients (89.0%). Mannitol and glycerin were frequently used. In addition, a high dose of steroid was administrated in most patients (87.7%) before BCT. Analgesics and sedatives were administrated in 11 patients before BCT. However, hypertonic saline was not used during the study period (Table 1).

**Table 1:** Baseline characteristics of barbiturate coma therapy.

Characteristic				Favorable neurological outcome (N = 25)	Poor neurological outcome (N = 48)	*p*-value
Age (yr)—median (IQR)				42.0 (31.0–52.0)	48.0 (38.5–58.0)	0.044
Gender, male—no. of patients (%)				14 (56.0)	23 (47.9)	0.512
BMI (kg/m^2^)—median (IQR)				24.4 (23.2–26.0)	23.0 (21.6–25.4)	0.051
Classification of brain tumor (Grading according to the 2016 WHO Classification)						0.124
	Meningiomas			13 (52.0)	14 (29.2)	
			Meningioma (I)	9 (36.0)	8 (16.7)	
			Atypical meningioma (II)	1 (4.0)	4 (8.3)	
			Anaplastic meningioma (III)	3 (12.0)	2 (4.2)	
	Diffuse astrocytic and oligodendroglial tumors			3 (12.0)	17 (35.4)	
			Diffuse astrocytoma (II)	0 (0)	2 (4.2)	
			Anaplastic astrocytoma (III)	0 (0)	1 (2.1)	
			Glioblastoma (IV)	3 (12.0)	14 (29.2)	
	Mesenchymal, non-meningothelial tumors			3 (12.0)	2 (4.2)	
			Hemangioblastoma (I)	3 (12.0)	2 (4.2)	
	Tumors of the sellar region			2 (8.0)	3 (6.2)	
			Craniopharyngioma (I)	2 (8.0)	1 (2.1)	
			Pituitary adenoma	0 (0)	2 (4.2)	
	CNS lymphoma			0 (0)	3 (6.2)	
	Chordoma			2 (8.0)	1 (2.1)	
	CNS metastasis			1 (4.0)	2 (4.2)	
	Others			1 (4.0)	6 (12.5)	
Origin of brain tumor						0.062
	Supratentorial			14 (56.0)	37 (77.1)	
	Infratentorial			11 (44.0)	11 (22.9)	
Pupillary reactivity						0.188
	Both reacted			17 (68.0)	22 (45.8)	
	One reacted			2 (8.0)	5 (10.4)	
	None reacted			6 (24.0)	21 (43.8)	
Started BCT time after ICU admission (hr)—median (IQR)				0.8 (0.3–16.2)	12.4 (0.4–49.6)	0.072
Started BCT immediately after tumor surgery				19 (76.0)	28 (58.3)	0.135
Early IICP management prior to BCT—no. of patients (%)						
	Invasive ICP monitoring					0.318
		External ventricular drain		8 (32.0)	19 (39.6)	
		Intraparenchymal or epidural ICP monitor		1 (4.0)	6 (12.5)	
	Decompressive craniectomy			4 (16.0)	8 (16.7)	0.999
	Lumbar drain			2 (8.0)	4 (8.3)	0.999
	Hyperosmolar agent					0.252
		Combination of mannitol and glycerin		13 (52.0)	29 (60.4)	
		Mannitol		7 (28.0)	14 (29.2)	
		Glycerin		0 (0)	2 (4.2)	
	Steroid			20 (80.0)	44 (91.7)	0.259
	Analgesics and sedatives			4 (16.0)	7 (14.6)	0.999
	Receipt of vasopressor or inotropics before BCT			0 (0)	10 (20.8)	0.013
Brain CT on BCT (Rotterdam score)				2.0 (1.0–3.0)	3.0 (2.0–3.0)	0.057
GCS on BCT—median (IQR)				3.0 (3.0–5.0)	3.0 (3.0–6.5)	0.669
APACHE II score on BCT—median (IQR)				18.0 (16.0–20.0)	19.0 (15.5–21.5)	0.414
SOFA score on BCT—median (IQR)				5.0 (4.0–6.0)	5.0 (4.0–7.0)	0.398

IQR, interquartile range; BMI, body mass index; CNS, central nerve system; WHO, World Health Organization; BCT, barbiturate coma therapy; ICU, intensive care unit; IICP, increased intracranial pressure; ICP, intracranial pressure; CT, computed tomography; GCS, Glasgow Coma Scale; APACHE, Acute Physiology and Chronic Health Evaluation; SOFA, Sequential Organ Failure Assessment

The clinical characteristics are shown in Table 2. Compared to patients showing favorable neurological outcome, those with poor neurological outcome were older, used vasopressors or inotropics before BCT, and underwent a longer duration of BCT and mechanical ventilation. Patients with poor neurological outcome also had higher serum glucose levels during the first 24 h after BCT, and glucose levels higher than 11.1 mmol/L were more common among patients with poor neurological outcome.

**Table 2:** Clinical characteristics and outcomes.

Characteristic				Favorable neurological outcome (N = 25)	Poor neurological outcome (N = 48)	*p*-value
Need for renal replacement therapy—no. of patients (%)				0 (0)	4 (8.3)	0.292
Need for invasive ICP monitoring—no. of patients (%)				21 (84.0)	39 (81.2)	0.999
Duration of BCT (days)—median (IQR)				3.1 (1.9–3.5)	3.8 (2.9–5.3)	0.016
Duration of mechanical ventilator (days)—median (IQR)				7.0 (5.0–10.0)	10.5 (8.0–16.0)	0.002
Duration of invasive ICP monitoring (days)—median (IQR)				6.0 (5.0–13.0)	8.0 (4.0–10.0)	0.963
Max dose of barbiturate (mg/kg/hr)—median (IQR)				3.0 (2.5–3.0)	3.0 (2.8–3.5)	0.386
Mean physiological values within 24hr after BCT (mm Hg)—median (IQR)						
	ICP			8.1 (5.2–10.6)	10.5 (6.7–14.4)	0.216
	CPP			84.7 (80.3–88.0)	80.1 (75.0–86.3)	0.068
	MAP			92.8 (88.5–99.0)	92.0 (86.7–97.1)	0.437
	CVP			6.3 (4.0–8.3)	6.0 (4.5–8.5)	0.839
Maximal ICP within 6hr after BCT (mm Hg)—median (IQR)				10.3 (7.0–14.0)	15.5 (11.0–23.6)	0.008
Use of vasopressor or inotripics						
	Norepinephrine			21 (84.0)	37 (77.1)	0.488
	Dopamine			16 (64.0)	23 (47.9)	0.191
Dose of vasopressor (μg/kg/min)—median (IQR)						
	Norepinephrine			0.2 (0.2–0.3)	0.2 (0.1–0.5)	0.338
	Dopamine			16.6 (10.3–21.9)	20.8 (14.0–28.8)	0.149
Steroid use—no. of patients (%)				25 (100)	48 (100)	
Hyperosmolar agent—no. of patients (%)						0.630
	Combination of mannitol and glycerin			20 (80.0)	42 (87.5)	
	Mannitol			2 (8.0)	1 (2.1)	
	Glycerin			2 (8.0)	4 (8.3)	
Complication of BCT						
	Organ dysfunction or failure					
		Acute lung injury or VAP		6 (24.0)	13 (27.1)	0.776
			Severe hypoxia	2 (8.0)	3 (6.2)	0.999
		Acute kidney injury		0 (0)	3 (6.2)	0.547
		Liver dysfunction		2 (8.0)	1 (2.1)	0.269
	Electrolyte imbalance					
		Hypernatremia (> 145 mmol/L)		16 (64.0)	33 (68.8)	0.682
		Hyponatremia (< 135 mmol/L)		4 (16.0)	6 (12.5)	0.727
		Hyperkalemia (> 5.0 mmol/L)		0 (0)	4 (8.3)	0.292
		Hypokalemia (< 3.5 mmol/L)		15 (60.0)	27 (56.2)	0.758
			Severe hypokalemia (< 2.5 mmol/L)	2 (8.0)	5 (10.4)	0.999
	Metabolic acidosis (pH < 7.35 & HCO3^-^ < 22 mmol/L)			3 (12.0)	14 (29.2)	0.100
	Hypotension (SBP < 90 mmHg)			20 (80.0)	38 (79.2)	0.933
	Infection			5 (20.0)	13 (27.1)	0.505
		Central nerve system infection		1 (4.0)	4 (8.3)	
		Pneumonia		4 (16.0)	8 (16.7)	
		Urinary tract infection		0 (0)	1 (2.1)	
		Arrhythmia		2 (8.0)	2 (4.2)	0.603
		Hypothermia		0 (0)	4 (8.3)	0.292
	Glucose level (mmol/L)					
		Max glucose level within 24hrs		8.9 (7.2–10.2)	10.8 (9.2–13.7)	0.004
		Glucose group within 24hr				0.018
			< 7.8	7 (28.0)	5 (10.9)	
			7.8–11.1	14 (56.0)	19 (41.3)	
			> 11.1	4 (16.0)	22 (47.8)	

ICP, intracranial pressure; BCT, barbiturate coma therapy; IQR, interquartile range; CPP, cerebral perfusion pressure; MAP, mean arterial pressure; CVP, central venous pressure; VAP, ventilator-associated pneumonia

The ICU mortality rate was 19.2 percent. The lengths of stay in the ICU and hospital were 14.9 (IQR 10.0–22.7) days and 54.7 (IQR 27.8–81.8) days, respectively. Survival to discharge was identified in 56 (76.7%) patients. Of these 56 survivors, 25 (34.2%) manifested favorable neurological outcomes (GOS of 4 or 5). The factors associated with BCT or complications after tumor surgery are listed in Table 3. Bleeding complications were the most common events during tumor surgery (Table 3).

**Table 3:** Factors associated with barbiturate coma therapy during tumor surgery.

Characteristic		Favorable neurological outcome (N = 25)	Poor neurological outcome (N = 48)	*p*-value
Factors associated with BCT after surgery				0.051
	Hemorrhage	9 (36.0)	20 (41.7)	
	Residual tumor	10 (40.0)	12 (25.0)	
	Worsening of brain edema	3 (12.0)	10 (20.8)	
	Tense brain parenchyma during or after surgery	3 (12.0)	4 (8.3)	
	Infarction	0 (0)	2 (4.2)	
Events during surgery				0.746
	Massive or sub-massive bleeding	6 (24.0)	7 (14.6)	
	Major vessel injury	2 (8.0)	3 (6.2)	
	Bleeding after tumor biopsy	1 (4.0)	3 (6.2)	

BCT, barbiturate coma therapy

### Intracranial pressure

**Fig 2:**
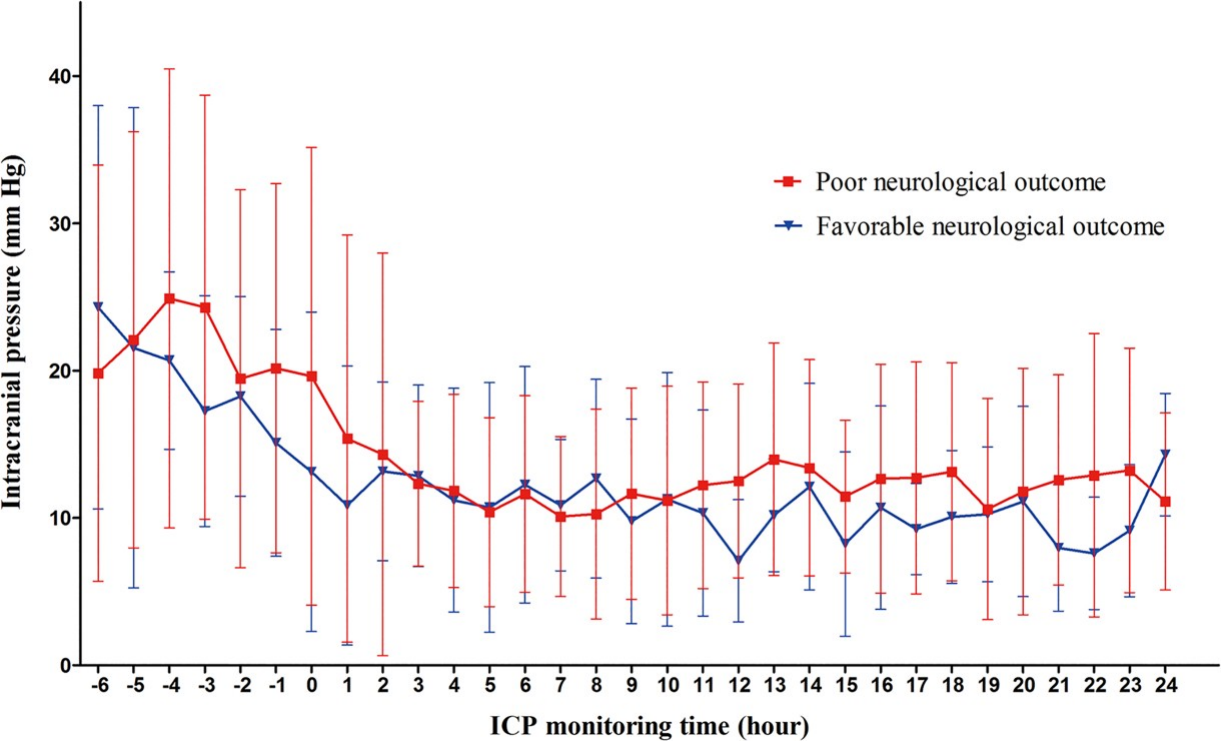
ICP patterns in 20 patients with established RICH before BCT, and monitored from 6 h before BCT. Four patients had favorable neurological outcome while 16 patients had poor neurological outcome. The ICP curves show the means ± standard deviation at each monitoring time. ICP, intracranial pressure; RICH, refractory intracranial hypertension; BCT, barbiturate coma therapy.

Invasive ICP monitoring was performed in 60 (82.2%) out of 73 patients. Twenty of these patients had RICH confirmed before BCT, and ICP was also monitored before and after BCT (Fig 2). In the remaining 40 patients, invasive ICP monitoring was started only after BCT. In the former 20 patients, ICP after BCT decreased significantly as compared to the pre-BCT levels. Further, the maximal ICP within 6 h after BCT (MI6) decreased significantly as compared to the pre-BCT level (16.2 [IQR 11.8–22.8] vs. 27.0 [IQR 23.0–39.0], *p* < 0.001). The mean ICP within 6 h after BCT also decreased significantly as compared to the pre-BCT values (11.7 [IQR 7.5–17.6] vs. 18.6 [IQR 14.4–31.9], *p* < 0.001).

Among the 60 patients monitored for ICP, the median MI6 was 13.2 (IQR 9.0–21.3) mm Hg. The MI6 in patients with favorable neurological outcome was significantly lower than that in patients with poor neurological outcome (10.3 [IQR 7.0–14.0] vs. 15.5 [IQR 11.0–23.6], *p* = 0.008).

**Fig 3:**
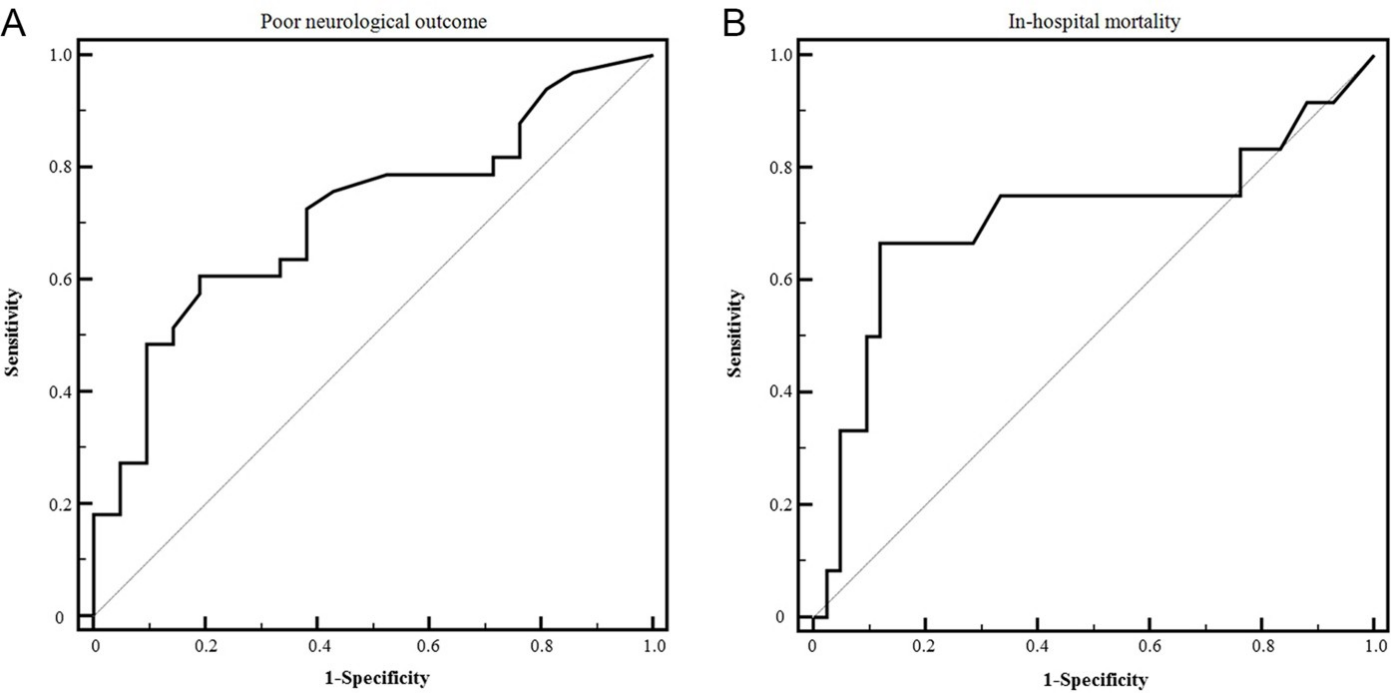
Receiver operating characteristic (ROC) curves for the prediction of clinical outcomes. ROC curves for the prediction of (A) poor neurological outcome and (B) hospital mortality associated with maximal intracranial pressure within 6 h after barbiturate coma therapy (n = 60).

**Fig 4:**
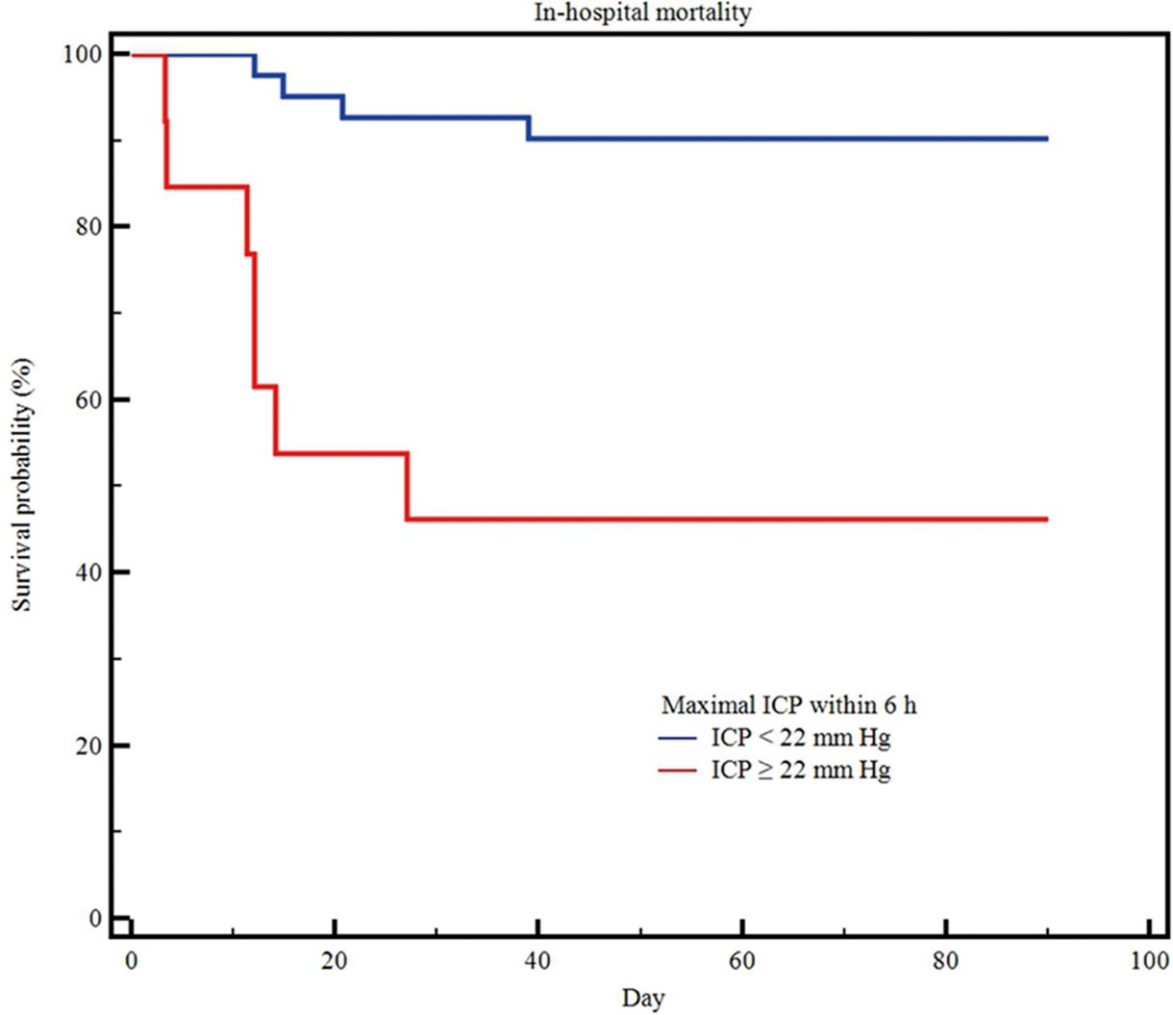
Kaplan-Meier three-month survival curves according to maximal intracranial pressure within 6 h after barbiturate coma therapy. Blue solid line, under 22 mm Hg; red solid line, over 22 mm Hg; adjusted hazard ratio 8.16, 95% CI, 2.373–28.056, *p* < 0.001, based on log-rank tests (n = 60).

Regarding the prediction of poor neurological outcome (Fig 3A), the MI6 resulted in an AUC of 0.715 (95% CI 0.576–0.830); A cut-off ≥15 mm Hg had a sensitivity of 60.6% (95% CI, 42.1% to 77.1%) and a specificity of 81% (95% CI, 58.1% to 94.6%). The MI6 among the survivors was also significantly lower than in non-survivors (11.8 [IQR 9.0–16.0] vs. 22.8 [IQR 11.0–33.0], p = 0.028). In the prediction of in-hospital mortality (Fig 3B), the M16 resulted in an AUC of 0.709 (95% CI 0.570–0.825). A cut-off ≥ 22 mm Hg had a sensitivity of 66.7% (95% CI, 34.9% to 90.1%) and a specificity of 88.1% (95% CI, 74.4% to 96.0%). The three-month mortality rate was significantly lower in patients with an MI6 < 22 mm Hg than in patients with an MI6 ≥ 22 mm Hg (9.8% vs. 53.8%, log-rank test, p < 0.001, Fig 4).

Multivariable logistic regression analysis revealed that supratentorial origin (adjusted OR 0.03, 95% CI 0.001–0.759), duration of mechanical ventilation (adjusted OR 1.41, 95% CI 1.115–1.787), and MI6 ≥ 15 mm Hg (adjusted OR 9.36, 95% CI 1.664–52.614) were significantly associated with poor neurological outcomes after BCT (Table 4). In addition, the use of vasopressor or inotropics before BCT (adjusted HR 11.94, 95% CI 2.546–56.034) and uncontrolled RICH (MI6 ≥ 22 mm Hg, adjusted HR 12.91, 95% CI 2.788–59.749) were significantly associated with in-hospital mortality (Table 4).

**Table 4:** Univariable and multivariable analyses of factors associated with clinical outcomes.

Characteristic				Poor neurological outcome		In-hospital mortality	
				Univariable analysis OR (95% CI)	Logistic regression OR (95% CI)	Univariable analysis HR (95% CI)	Cox regression HR (95% CI)
Clinical factor							
	Age			0.33 (0.999–1.081)		0.08(0.986–1.073)	
	Gender, male			1.64 (0.524–3.656)		0.28 (0.407–3.583)	
	Origin of brain tumor (supratentorial)			0.38 (0.134–1.068)	0.03 (0.001–0.759)	0.42 (0.107–1.633)	
Factor associated with BCT							
	Receipt of vasopressor or inotropics before BCT			6.04 (0.494–0.737)		4.25 (1.059–17.063)	11.94 (2.546–56.034)
	Maximal dose of norepinephrine during BCT			4.40 (0.382–50.705)		12.91 (1.265–131.675)	
	Duration of BCT			1.46 (1.066–2.001)		1.04 (0.827–1.307)	
	Duration of mechanical ventilator			1.20 (1.051–1.374)	1.41 (1.115–1.787)	1.03 (0.966–1.094)	
	Maximal ICP within 6hr after BCT						
		≥ 15 mm Hg		6.38 (1.572–25.852)	9.36 (1.664–52.614)	5.00 (1.265–19.762)	
		Uncontrolled RICH (≥ 22 mm Hg)		4.75 (0.934–24.168)		14.80 (3.235–67.719)	12.91 (2.788–59.749)
	CPP mean with 24hr			0.94 (0.872–1.004)		0.93 (0.857–0.997)	
Laboratory findings							
	Glucose level						
		Max glucose level within 24hrs		1.01 (0.999–1.017)		0.99 (0.991–1.007)	
		Glucose group within 24hr (mmol/L)					
			< 7.8	1 (reference)		1 (reference)	
			7.8–11.1	1.90 (0.498–7.251)		4.13 (0.464–36.702)	
			> 11.1	7.70 (1.609–36.860)		4.05 (0.439–37.425)	

OR, odd ratio; CI, confidence intervals; HR, hazard ratio; BCT, barbiturate coma therapy; ICP, intracranial pressure; RICH, refractory intracranial hypertension; CPP, cerebral perfusion pressure

### Complications of BCT

Hypotension (79.5%) was the most common complication during BCT. Acute lung injury and ventilator-associated pneumonia (26.0%) were the most common organ dysfunctions during BCT. However, only five (6.8%) patients exhibited severe hypoxia. In 18 (24.7%) patients, infectious complication and pneumonia (16.4%) were the most common infections. There were no significant differences in complications associated with BCT between the two groups with different neurological outcomes (Table 2). No significant differences were detected in complications between survivors and non-survivors. No BCT-induced death was observed.

## Discussion

In the present study, we evaluated which predictors are associated with clinical outcomes when BCT is used as a second-tier therapy to control RICH after brain tumor surgery. The major findings of this study were as follows: 1) ICPs after BCT were significantly decreased as compared with ICPs before BCT in patients with RICH. 2) The survival rate of patients who underwent BCT after brain tumor surgery was relatively good. After tumor surgery, three-fourths of patients who underwent BCT survived. Furthermore, half of all survivors showed favorable neurological outcome. 3) The early control of ICP after BCT was associated with good clinical prognosis. Controlled RICH (ICP < 22 mm Hg) was an important predictor of survival after BCT, and ICP < 15 mm Hg within 6 h of BCT was particularly associated with favorable neurological outcome. 4) A low incidence of fatal complications after BCT was observed. In addition, there were no significant differences in BCT-induced complications between the two groups with different neurological outcomes.

Since the 1930s, high-dose barbiturate has been known to decrease ICP [18]. The mechanism of the reduction of ICP by barbiturates is based on three theories. First, barbiturates induce vasoconstriction in normal brain areas (shunting blood to ischemic brain tissue) and reduce ICP [18,19]. Second, barbiturates decrease metabolic oxygen demand with an accompanying reduction of cerebral blood flow, with an immediate effect on ICP [3,18,20]. Barbiturates may exert protective effects via the stabilization of lysosomal membrane, reduction of intracellular calcium concentration, modification of amino acid and neurotransmitter release, scavenging of free radicals, alteration of fatty acid metabolism, reduction in cerebrospinal fluid production, membrane stabilization, and suppression of seizure [18,21–24]. RICH can be controlled with high-dose barbiturate treatment [3–5,18]. However, almost all studies on BCT have evaluated patients with TBI and malignant infarction [4–6,25–27], and limited data are available on patients with brain tumor. In this study, ICPs after BCT were also significantly decreased as compared to ICPs before BCT in RICH patients undergoing tumor surgery.

Uncontrolled RICH is associated with mortality in TBI patients [5,6]. The mortality rate in TBI patients with severe RICH exceeds 80% when the ICP cannot be reduced to less than 20 mmHg [4]. In this study, uncontrolled RICH was also associated with mortality in patients who underwent brain tumor surgery. However, limited data are available regarding the neurological outcomes associated with BCT or the target level of ICP for favorable neurological outcome [5,6]. In this study, although controlled RICH was associated with survival in patients who underwent tumor surgery, more tightly controlled ICP < 15 mm Hg within 6 h of BCT was particularly associated with favorable neurological outcome. Therefore, the aggressive management of intracranial hypertension to normal ICP in the early stages of BCT may be necessary for optimal neurological outcomes.

In previous studies, the complications occurring during BCT included hypotension in 58% of patients, hypokalemia in 82%, respiratory complications in 76%, infections in 55%, hepatic dysfunction in 87%, and renal dysfunction in 47% [20,28]. However, in this study, the incidence of BCT-induced complications was relatively lower than those reported by these previous studies [20,28]. Although acute lung injury was the most common organic dysfunction during BCT, severe hypoxia was observed in a few patients in this study as well. In addition, no significant increase was observed in the incidence of BCT-induced complications associated with neurological outcome and mortality.

In this study, the survival rate of patients who underwent BCT after brain tumor surgery was relatively good, though only half of survivors showed favorable neurological outcome. BCT was performed by experienced nursing staff using the critical pathway [15]. By verifying the clinical validity of the critical pathway, visible effects were obtained by standardizing the treatment pathway for patients with RICH in our previous study [15]. Eventually, BCT-induced complications were reduced through protocol-based therapy and skilled nursing care. In addition, the quality of supportive care in critically ill patients was improved as compared with previous studies. For the supportive care of patients undergoing BCT after brain tumor surgery, neurointensivist co-management was conducted. A neurointensivist was involved in general critical management, including hemodynamic monitoring; nutritional support; and the use of mechanical ventilation, renal replacement therapy, etc [29]. Therefore, these factors might have led to the better clinical outcomes of BCT than those reported in previous studies [6,20,27,28].

This study has several limitations. First, the GOS was retrospectively determined based on medical records. Second, the nonrandomized nature of the registry data may have resulted in selection bias. Third, invasive ICP monitoring was performed in a limited number of patients before BCT. Fourth, the CT findings were compared with those of BCT using the Rotterdam CT scan scoring system. However, this scoring system has generally been used for TBI patients. We used this scoring system because there is no CT scoring system available for evaluating the severity of brain damage after tumor surgery. Hypertonic saline was not administrated during the study period. We did not determine the target level of serum sodium. In addition, therapeutic hypothermia was not performed during the study period. Therefore, if hypertonic saline or therapeutic hypothermia was used, it could have affected the results. Finally, our study has limited statistical power due to the small sample size. Although it still provides valuable insight, prospective large-scale studies are needed in the future to evaluate the usefulness of BCT and the predictors of neurological outcomes in patients with brain tumor surgery to arrive at evidence-based conclusions.

## Conclusions

In this study, we found that BCT is a treatment modality with acceptable safety and may thus be a reasonable choice as a last resort to control RICH in patients after brain tumor surgery. The active and timely control of RICH may be beneficial for patients’ outcomes.
